# Utilization of TREC and KREC quantification for the monitoring of early T- and B-cell neogenesis in adult patients after allogeneic hematopoietic stem cell transplantation

**DOI:** 10.1186/1479-5876-11-188

**Published:** 2013-08-14

**Authors:** Angela Mensen, Christoph Ochs, Andrea Stroux, Friedrich Wittenbecher, Martin Szyska, Luisa Imberti, Simon Fillatreau, Lutz Uharek, Renate Arnold, Bernd Dörken, Andreas Thiel, Carmen Scheibenbogen, Il-Kang Na

**Affiliations:** 1 Institute of Medical Immunology, Charité CVK, Berlin, Germany; 2 Institute for Biometry and Clinical Epidemiology, Charité CBF, Berlin, Germany; 3 Department of Hematology, Oncology and Tumor Immunology, Charité, Berlin, Germany; 4 Experimental and Clinical Research Center (ECRC), Berlin, Germany; 5 Laboratorio Interdipartimentale di Biologia Cellulare e Radio-Biologia, Brescia, Italy; 6 German Rheumatism Research Center, Leibniz Institute, Berlin, Germany; 7 Regenerative Immunology and Aging, Berlin-Brandenburg Center for Regenerative Therapies (BCRT), Charité CVK, Berlin, Germany; 8 Berlin-Brandenburg Center for Regenerative Therapies (BCRT), Charité CVK, Berlin, Germany

**Keywords:** Allogeneic hematopoietic stem cell transplantation, Acute leukemia, Simultaneous TREC/KREC quantification assay, Monitoring immune reconstitution

## Abstract

**Background:**

After hematopoietic stem cell transplantation (HSCT) T- and B-cell reconstitution from primary lymphoid organs are a prerequisite for an effective early lymphocyte reconstitution and a long-term survival for adult patients suffering from acute leukemia. Here, we asked whether quantification of T cell receptor excision circle, (TREC) and kappa-deleting recombination excision circle (KREC) before and within six month after allogeneic HSCT could be used to measure the thymic and bone marrow outputs in such patients.

**Methods:**

We used a duplex real time PCR assay to quantify the absolute copy counts of TREC and KREC, and correlated the data with absolute cell counts of CD3^+^CD4^+^ T-cell and CD19^+^ B-cell subsets determined by flow cytometry, respectively.

**Results:**

By comparing two recently proposed naïve T cell subsets, CD31^+^ naive and CD31^-^ naive T cells, we found a better correlation for the CD31^+^ subset with TREC level post alloHSCT, in line with the assumption that it contained T cells recently derived from the thymus, indicating that TREC levels reflected real thymic *de novo* production. Transitional as well as naïve B cells highly correlated with KREC levels, which suggested an association of KREC levels with ongoing bone marrow B cell output. CD45RO^+^ memory T cells and CD27^+^ memory B cells were significantly less correlated with TREC and KREC recovery, respectively.

**Conclusion:**

We conclude that simultaneous TREC/ KREC quantification is as a suitable and practicable method to monitor thymic and bone marrow output post alloHSCT in adult patients diagnosed with acute leukemia.

## Background

Allogeneic hematopoietic stem cell transplantation (alloHSCT) is a common treatment strategy to cure hematological malignancies and other hematological disorders [1]. Pretransplant conditioning therapy providing space for stem cell engraftment as well as immune suppression therapy to prevent transplant rejection are usually associated with a profound long-term humoral and cellular immune deficiency [2,3]. The development of graft-versus-host disease (GvHD) can further exacerbate lymphocytopenia thereby increasing the risk of life-threatening viral, bacterial and fungal infections [4,5]. T- and B- cell reconstitution starts about 3–6 months after alloHSCT representing a mixture of peripheral naïve and memory cell expansion as well as *de novo* development from primary lymphoid organs [2,6-9]. While expansion of mature T and B cells will only provide a transient immune protection and is likely to be repertoire restricted as well as potentially allo-reactive, the *de novo* production from hematopoietic stem cells is important for long-term immune protection and tolerance [2,6,7,10,11]. For individualized monitoring of immune reconstitution in the clinic, it is important to have validated methods to track T- and B-cell reconstitution after alloHSCT in the relevant patient groups.

A method to measure thymic output in the context of aging, immunodeficiency and HSCT is the quantification of T cell receptor excision circles (TRECs) [7,12-19]. TRECs are stable episomal, non-replicative DNA circles generated during T cell receptor (TCR) alpha chain rearrangement when the excision of the D locus occurs [20]. About 70% of all newly produced T cells are TREC positive. TREC level are elevated in T cell preparations of children and then decline with increasing age due to thymic involution [12]. Nevertheless, TREC levels can still be detected in elderly people indicating that the thymus partly retains its function in old age [12,21].

In the context of HSCT, the thymus was shown to substantially contribute to T-cell immune reconstitution after HSCT [21], and positive correlations between TREC and total naïve CD4^+^ T-cell counts were assessed in adult and pediatric alloHSCT patients [7,15,16]. Recently, CD31 was introduced as a marker to distinguish naïve T cells into recently thymic-derived CD4^+^CD31^+^ naïve T cells and more mature recirculating CD4^+^CD31^-^ naïve T cells in the peripheral blood [22]. CD4^+^CD31^+^ naïve T cells have a higher TREC content and broader TCR repertoire than CD4^+^CD31^-^ naïve T cells. Clinical studies found a positive correlation between TREC content and frequency of CD4^+^CD31^+^ naïve T cells in adult autologous and pediatric allogeneic HSCT patients [17,19]. However, these studies did not investigate CD4^+^CD31^-^ naïve T cells, excluding the possibility of comparing the correlation of TRECs with these two subsets of naïve T cells in these patients. Since the regulation of CD31 expression on T cells is not known in this clinical context, the validity of this receptor as a marker of recent thymic emigrants after HSCT remains unresolved. Such comparison is also missing in adult alloHSCT patients, which present with different clinical features than the situations described above*.* Importantly, consideration of CD31 expression as a marker for recent thymic emigrants needs to take into account that “old” naïve T cells could maintain CD31 expression regardless of the time they egressed the thymus.

It recently became possible to simultaneously measure the thymic T- and bone marrow B-cell output using a duplex real-time PCR for quantification of TRECs and κ-deleting recombination excision circles (KRECs), which are generated during B cell development as a result of light chain rearrangements [13,23]. Similarly to TRECs, KRECs are progressively diluted as B cells proliferate and mature in the periphery [23]. KRECs are randomly found in about 50% of newly produced B cells. Studies in children post alloHSCT reported an increase in KREC level concomitant with B cell recovery [13,18]. However, these studies investigated total blood B cells without considering their heterogeneity. In blood, B cells can be divided into immature transitional B cells, which recently emigrated from the bone marrow, naïve B cells, and memory B cells [23]. In the context of HSCT, correlations between KREC levels and different B cell subsets remain to be compared.

The aim of our study was to evaluate the suitability of TREC and KREC quantification as a marker to monitor T and B cell reconstitution in adult alloHSCT patients. For this, we simultaneously correlated absolute TREC and KREC copy counts with different flow cytometric determined peripheral blood T- and B-cell subsets. Here, we focused on very early time points after transplantation as an effective lymphopoiesis during the first months after transplantation was shown to be associated with improved overall survival, thus emphasizing the importance of immune monitoring during that time period [16,24].

## Methods

### Patients

The study included 15 adult patients diagnosed with hematological malignancies who received full intensity (n=8) or reduced intensity (n=7) conditioning prior to allogeneic HSCT according to standard protocols. Clinical characteristics of these patients are listed in Table 1. The stem cells were obtained from granulocyte-colony stimulating-factor (G-CSF) treated HLA-matched-related (MRD, n=5) or unrelated (MRD, n=10) healthy donors. GvHD prophylaxis included Cyclosporine A in combination with methotrexate or mycophenolate mofetil medication as well as ATG treatment in case of MUD transplantation according to standard protocols. For the kinetic study fresh patient blood samples were obtained once before transplantation and at day 15, 30, 60, 90 and 180 post transplantation and were immediately used for analysis. The study was approved by the Charité-Berlin local ethics committee (no. EA4/128/09 and no. EA1/233/09) and patients signed an informed consent.

**Table 1:** Clinical characteristics of alloHSCT patients

	**n=15**	**%**
**Median age (range)**	**57 (28–68)**	
<50	5	30
>50	10	70
Sex		
female	8	53
male	7	47
Diagnosis		
AML	13	87
ALL	2	13
Transplant		
MUD	10	70
MRD	5	30
Conditioning		
full intensity	8	53
47 reduced intensity	7	47
GvHD (acute = + Chronic)		
yes	16	89
no	2	11

*AML*, acute myeloid leukemia; *MUD*, matched unrelated donor; *MRD*, matched related donor; *GvHD*, graft-versus-host disease.

### Flow cytometry analysis of lymphocyte subpopulations

Peripheral blood mononuclear cells (PBMCs) were isolated by density gradient centrifugation (Ficoll-Hypaque; GE Healthcare) from fresh heparinized patient blood samples. After washing with PBS the cells were stained in two staining panels for 20 min at 4°C with the following fluorochrome-conjugated anti-human monoclonal antibodies: T cell panel - CD3 APC/Cy7 (clone UCHT1), CD4 AlexaFluor 700 (clone RPA-T4), CD45RA PerCP-Cy5.5 (clone HI100), CD45RO Pacific Blue (clone UCHL1), CD62L APC (clone DREG-56), CD31 PE (clone WM59), CD197 (CCR7) Alexa Fluor 488 (clone TG8/CCR7) from Biolegend and CD27 PE-Cy7 (clone O323) from eBiosciences; B cell panel - CD19 Pacific Blue (clone HIB19), IgD-FITC (clone IA6-2), CD27 PerCP-Cy5.5 (clone O323), CD38 PE-Cy7 (clone HIT2) from Biolegend. PBMCs were washed and analyzed by flow cytometry using a BD™ LSRII flow cytometer (Becton Dickinson, Palo Alto, CA, USA) supported by FlowJo 9.3 software (TreeStar, Ashland, OR, USA).

### Real-time PCR for TREC and KREC quantification

DNA was purified from PBMCs using the QIAamp DNA Blood Mini Kit according the manufacturer’s instructions (Qiagen, Hilden, Germany). For the simultaneous detection of TREC and KREC copy numbers a duplex Real-Time PCR assay described by Sottini *et al.*[13] was performed on the 7500 Real-Time PCR System (Applied Biosystems, Foster City, CA, USA). Accordingly, the following primer and probes were used: SJ TREC forward primer (5’-CAC ATC CCT TTC AAC CAT GCT-3’) and reverse primer (5’-TGC AGG TGC CTA TGC ATC A-3’) with the probe (5’-FAM-ACA CCT CTG GTT TTT GTA AAG GTG CCC ACT TAMRA-3’) and SJ KREC forward primer (5’-TCC CTT AGT GGC ATT ATT TGT ATC ACT-3’) and reverse primer (5’-AGG AGC CAG CTC TTA CCC TAG AGT-3’) with the probe (5’-HEX-TCT GCA CGG GCA GCA GGT TGG-TAMRA-3’). For the housekeeper gene TRAC (T cell receptor alpha constant gene) the forward primer (5’-TGG CCT AAC CCT GAT CCT CTT-3’) and reverse primer (5’-GGA TTT AGA GTC TCT CAG CTG GTA CAC-3’) with the probe (5’-FAM-TCC CAC AGA TAT CCA GAA CCC TGA CCCTAMRA- 3’) were used. PCR reactions were developed in MicroAmp®Optical 96-well reaction plates (Applied Biosystems) in a final volume of 25 μl consisting of 5 μl (100-500 ng) genomic DNA, 12.5 μl 2xTaqMan Universal PCR master mix containing AmpErase UNG (Applied Biosystems) and primers and probes for TREC, KREC and TRAC at a final concentration of 900 and 200 nM, respectively. The PCR setup was as follows: 1x (2 min 50°C), 1x (10 min 95°C), 45x (15 sec 95°C, 1 min 60°C). TREC, KREC and TRAC copy number were determined by extrapolating the values from a unique standard curve which was obtained by the amplification of serial dilutions of a triple-insert plasmid (10^6^, 10^5^, 10^4^, 10^3^, 10^2^, 10^1^) , which encodes one gene copy of TREC, KREC and TRAC each [13]. The unique standard curve enabled the elimination of variability related to direct DNA quantificaton. Assessment of TRAC served as a control for the quality and quantity of genomic DNA in the sample. The number of TREC or KREC copies per ml blood was calculated with the following formula:

$$\begin{array}{ll} \frac{\mathrm{mean}\;\mathrm{KREC}\;\mathrm{or}\;\mathrm{TREC}\;\mathrm{quantitiy}}{\mathrm{mean}\;\mathrm{of}\;\mathrm{TRAC}\;\mathrm{quantitiy}/2}\times & \;\left(\mathrm{lymphocyte}\right)\\ & \;\left(+\mathrm{monocyte}\;\mathrm{count}\right)/\mathrm{ml} \end{array}$$

The mean of TRAC quantity was divided by two considering the presence of two TRAC gene copies per cell. True count beads (BD Biosciences) were added to whole blood samples of patients to determine the absolute lymphocyte and monocyte count per ml blood.

### Statistics

Clinical characteristics are presented as absolute and relative frequencies. In order to account for the intra-individual dependency over time of the TREC and KREC data and the corresponding T and B cell subsets data, GEE (generalized estimating equations) analyses [25] have been performed to calculate standardized regression coefficient betas (β) and p-values. The β coefficient measures the strength of the effect of the respective independent variable on the dependent variable that means the higher the β, the stronger the association between the two measurements. The standardized β values were used to achieve independency of the measure units. Additionally, logarithmic transformations have been performed to obtain normality. Two-sided p-values ≤ 0.05 are considered statistically significant. No Bonferroni correction has been performed. All statistical analyses were done using the commercially available software SPSS 20.

## Results and discussion

### Molecular and flow cytometric assessment of T and B cell reconstitution in alloHSCT patients

Here, we simultaneously assessed the correlation between TREC/ KREC levels and different T-/ B-cell subsets in adult patients after alloHSCT (Table 1), with the aim to test whether this duplex real time PCR assay could be applied for monitoring of T- and B-cell neogenesis in this particular clinical context. This duplex PCR assay allows the quantification of TREC and KREC in the same PCR reaction. Thereby, naïve T and B cells emigrating from the thymus and bone marrow, respectively, can be monitored simultaneously without any additional expenses. Since T and B cell maturation/differentiation are organ-specific and exhibit individual kinetics, more information about the immune cell reconstitution can be achieved by using TREC and KREC.

**Figure 1:**
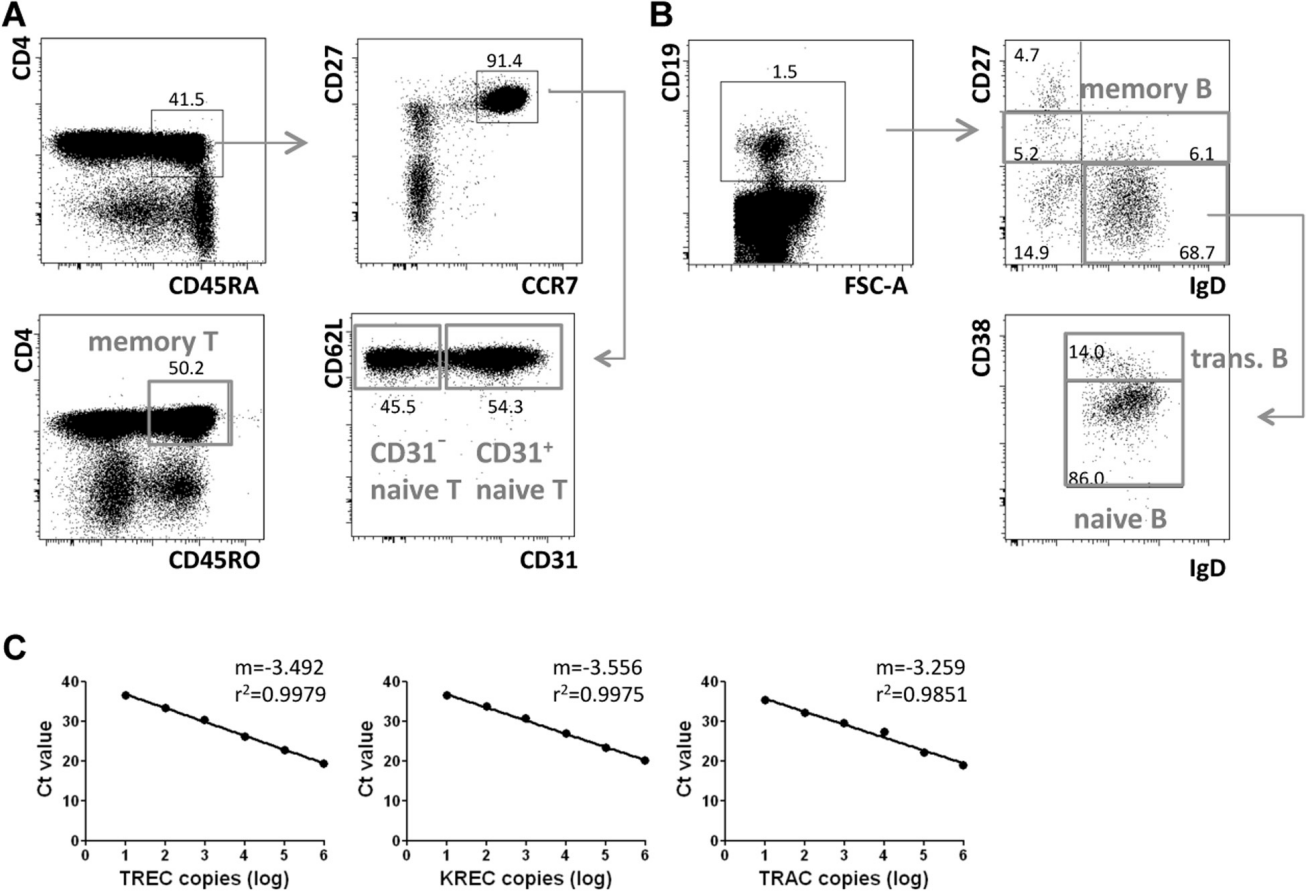
**Flow cytometric identification of human CD3**^**+**^**CD4**^**+ **^**T- and CD19**^**+ **^**B-cell subsets for correlation with absolute TREC and KREC quantity post alloHSCT. (A)** Human peripheral blood CD3^+^CD4^+^ T-cell subsets were defined as CD45RA^+^CCR7^+^CD62L^+^CD27^+^CD31^+^ naïve T cells, CD45RA^+^CCR7^+^CD62L^+^CD27^+^CD31^-^ naïve T cells and CD45RO^+^ memory T cell cells. **(B)** Within the CD19^+^ B cell compartment, B-cell subsets were identified by gating on IgD^+^CD27^-^CD38^hi^ transitional B cells, IgD^+^CD27^-^CD38^int^ naïve B cells and CD27^+^ memory B cells. Dotplots in **A** and **B** are representative of a patient at day 90 post alloHSCT. Numbers indicate percentages of gated cell subsets. **(C)** Absolute TREC, KREC and TRAC copy numbers were extrapolated from standard regression lines obtained by the determination of Ct values for the serial diluted triple insert plasmid encoding one copy of TREC, KREC and TRAC. Shown is one representative standard line for each gene. m indicates the slope of the linear regression line and r^2^ the correlation between linear regression line and obtained Ct-values as a marker for the accuracy of the respective standard dilutions.

Within CD3^+^ T cells, absolute cell counts of CD4^+^CD45RA^+^CD31^+^ and CD4^+^CD45RA^+^CD31^-^ naïve, as well as CD4^+^CD45RO^+^ memory T cells per ml blood were determined by flow cytometry (Figure 1A). Herein, CD4^+^CD45RO^+^ memory T cells included CCR7^+^ central memory and CCR7^-^ effector memory T cells, but excluded the CD4^+^CD45RA^+^CD27^-^CD127^+^ effector memory subpopulation due to a lack of CD127 in our staining. CD19^+^ B-cell neogenesis was analysed for the same patients by determining absolute cell counts of IgD^+^CD27^-^CD38^hi^ transitional B cells, IgD^+^CD27^-^CD38^int^ naïve B cells and CD19^+^CD27^+^ memory B cells per ml blood (Figure 1B). In parallel analyses, we determined absolute TREC and KREC counts from total peripheral blood mononuclear cells (PBMCs) as copies per ml blood. By determining the copy number per blood volume and not per cell, we bypassed the potential influence of peripheral proliferation on TREC or KREC numbers [26]. Absolute copy numbers of TREC, KREC and the housekeeping gene TRAC were obtained by extrapolation from standard regression lines determined for each gene during each qRT-PCR reaction (Figure 1C). Standard regression lines usually were of high quality with correlation coefficients of nearly r^2^=1. The number of patient samples included in our analysis depended on either availability of samples at defined time points after alloHSCT or the detection limit of the TREC/KREC assay. We included only DNA samples with a minimum concentration of 50ng/ml as lower concentrations were outside of linearity of the standard curve and therefore appeared not to be qualified to test reliable TREC/KREC values. Especially during neutropenia cell numbers were limiting.

To determine the specificity of the TREC/KREC quantification we performed the assay on genomic DNA from the human erythroleukemia cell line K562 and from the human T cell leukemia cell line Jurkat (Additional file 1: Table S1). Both cell lines are negative for TREC [27,28] and KREC probably due to clonal proliferation, but positive for TRAC. Accordingly, we found an amplification of TRAC but no off-target amplification of TREC and KREC on these cell lines Genomic DNA from PBMCs of a healthy donor served as a positive control.

### TREC and T cell subset recovery after alloHSCT

**Figure 2:**
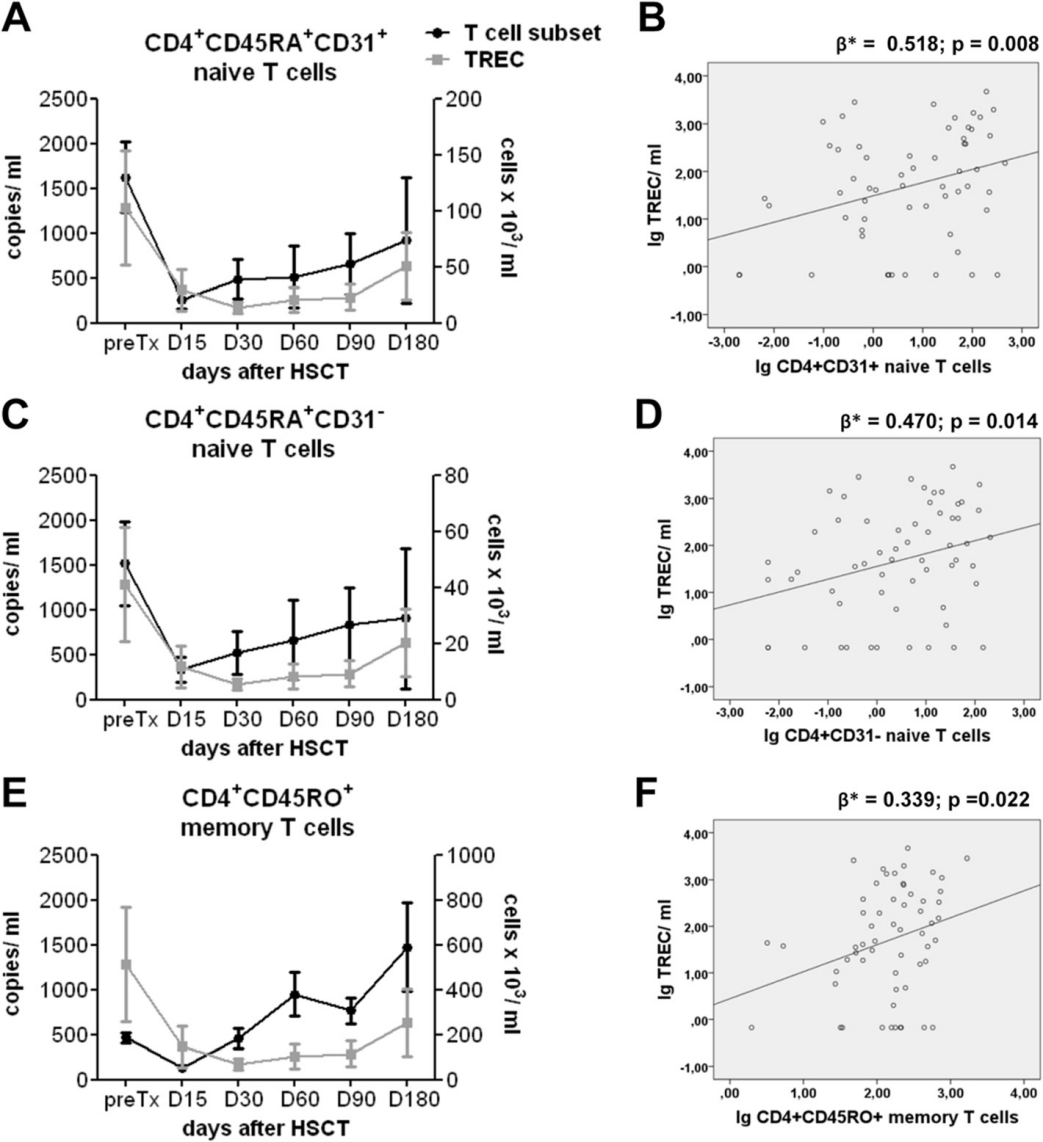
**Correlation analysis of TREC quantification with T-cell subset recovery after alloHSCT. (A,C,E)** PBMCs of adult alloHSCT patients were analyzed for absolute CD4^+^CD3^+^ T-cell subset counts by flow cytometry and absolute TREC copy number by quantitative RT-PCR before (preTx) and after transplantation at the indicated time points (preTx n=7, D15 n=11, D30 n=13, D60 n=8, D90 n=13, D180 n=8). Mean values of TREC copy number per ml blood (grey line, **A,C,E**) and absolute number of CD31^+^ naive **(A)**, CD31^-^ naïve **(C)** and memory T cells **(E)** per ml blood (black line) ± standard error of the mean (SEM) are shown. **(B,D,F)** Absolute TREC copy number and T-cell subset count are shown as scatterplot depiction with linear regression line for CD31^+^ naive **(B)**, CD31^-^ naïve **(D)** and memory T cells **(F)**. The regression coefficients beta (β*) and p-values are displayed for each subpopulation.

Absolute cell counts of CD4^+^CD45RA^+^CD31^+^ naïve, CD4^+^CD45RA^+^CD31^-^ naïve and CD4^+^CD45RO^+^ memory T cells were determined by flow cytometry before and within six month after transplantation. Absolute numbers of CD4^+^CD45RA^+^CD31^+^ and CD4^+^CD45RA^+^CD31^-^ naïve T cells were characterized by slow reconstitution kinetics, which started to increase on day 30, and remained below pre-transplant level until day 180 (mean 130±32 cells/ml and 49±15 cells/ml, respectively) (Figure 2A,C). The strongest expansion was seen for memory T cells that reached pre-transplant levels (mean 186±20 cells/ml) already on day 30, and then increased further (589±196 cells/ml on day 180) (Figure 2E). Such initial expansion of memory T cells within the first six months post alloHSCT was similarly described in previous publications and was shown to represent antigen-driven immune activation with increased susceptibility to activation-induced cell death of alloreactive donor-derived memory T cells [15].

In parallel analyses, we determined absolute TREC counts from total peripheral blood mononuclear cells (PBMCs) as copies per ml blood. TRECs drastically declined after transplantation before recovering around day 180 (Figure 2A,C,E). At day 180, TRECs were still below the pretransplant level (mean 1285±634 copies/ml). TREC values of 2306 copies per ml were reported for healthy controls [14].

After having quantified amounts of distinct T cell subsets and TRECs at various time points after alloHSCT, we then tested the possible correlation between these parameters (Figure 2B,D,F). We determined standardized β-values by perfoming GEE analyses that account for longitudinal, i.e. intraindividually dependent data. Since in our study we consider the entire information of all measurement time points, the GEE analysis is most appropriate to model the longitudinal character of the data accurately. In contrast to these point estimates usual linear regression models would have been underestimated and thus been too optimistically. We observed that in line with previous publications, TREC recovery correlated with the numbers of both, CD4^+^CD45RA^+^CD31^+^ and CD4^+^CD45RA^+^CD31^-^ naïve T cells [7,15,17,19]. A better correlation was observed for CD4^+^CD45RA^+^CD31^+^ (Figure 2B) than for CD4^+^CD45RA^+^CD31^-^ (Figure 2D) naïve T cells (ß = 0.518, p < 0.01 for CD4^+^CD45RA^+^CD31^+^ naïve; ß = 0.470, p < 0.05 for CD4^+^CD45RA^+^CD31^-^ naïve). This is consistent with the notion that CD4^+^CD45RA^+^CD31^+^ naïve T cells are more likely recent thymic emigrants, while CD4^+^CD45RA^+^CD31^-^ naïve T cells contain more mature cells that resided in the periphery for longer times and proliferated [22], even though this had not been tested so far in the context of alloHSCT. Lymphocytopenia after conditioning therapy and T cell-depletion drives cell proliferation in the “empty” host especially within the first weeks post transplantation when IL-7 serum levels are increased, leading to a subsequent loss of TRECs [15,29]. However, the existence of “old” naïve T cells with maintained CD31 expression can not be excluded, so that CD31 expression does not necessarily represent an exclusive marker for T cells that recently emigrated from the thymus. [30] The poorest correlation was found for the CD4^+^CD45RO^+^ memory T cell subset (Figure 2F) (ß = 0.339, p < 0.05). This is in line with observations made in healthy individuals, whose memory T cells exhibited a ten-fold lower TREC content than naïve T cells [31].

Immune reconstitution differs in patients receiving full-conditioning and reduced-intensity conditioning therapy. For example, depending on the level of toxicity variable damage/ destruction of the bone marrow niche or thymus as well as of the host hematopoietic cell compartment occurs. Consequently, bone marrow and thymus output is more impaired after high-intensity conditioning therapy, whereas expansion of host T cells contributes more to the T cell repertoire after reduced-intensity conditioning therapy. We therefore separated patients receiving either full-conditioning or reduced-conditioning, and could still observe that TREC levels correlated with CD31^+^ naïve CD4 T cells (Additional file 2: Figure S1A).

More patients, preferably independent groups of patients, are required to estimate significant differences of β values between each of these T cell subsets and in dependency on different clinical parameters, which should definitely be analyzed in future studies in order to affirm this assay.

Recently, protein tyrosine kinase 7 (PTK7) as a novel marker for human CD4^+^ recent thymic emigrants and the assay to determine the sj-TREC / β-TREC ratio for thymic function assessment have been introduced [27,32]. PTK7+ naïve CD4+ recent thymic emigrants contain a higher level of TRECs, are more responsive to interleukin 7 and rapidly decrease after thymectomy. Although promising, this marker has not been applied in the setting of HSCT. Interestingly, expression of PTK7 was also found on AML blasts [33], so value of PTK7 as a recent thymic emigrant marker post transplantation in AML patient should be evaluated [27]. The sj-TREC / β-TREC ratio reflects the intensity of proliferation between DβJβ rearrangement and the VαJα rearrangement, which in turn is the major determinant of the number of recent thymic emigrants produced. The major advantages of this technology are independence of peripheral proliferation, measurement of thymic neogenesis of all αβ-T cells independent of their phenotype and gain of information about T cell receptor diversity [32]. However, this powerful methodology is, in contrast to the duplex PCR assay presented here, labor intensive, even in the simplified approach presented by Ferrando-Martinez et al. [34].

### KREC and B cell subset recovery after alloHSCT

**Figure 3:**
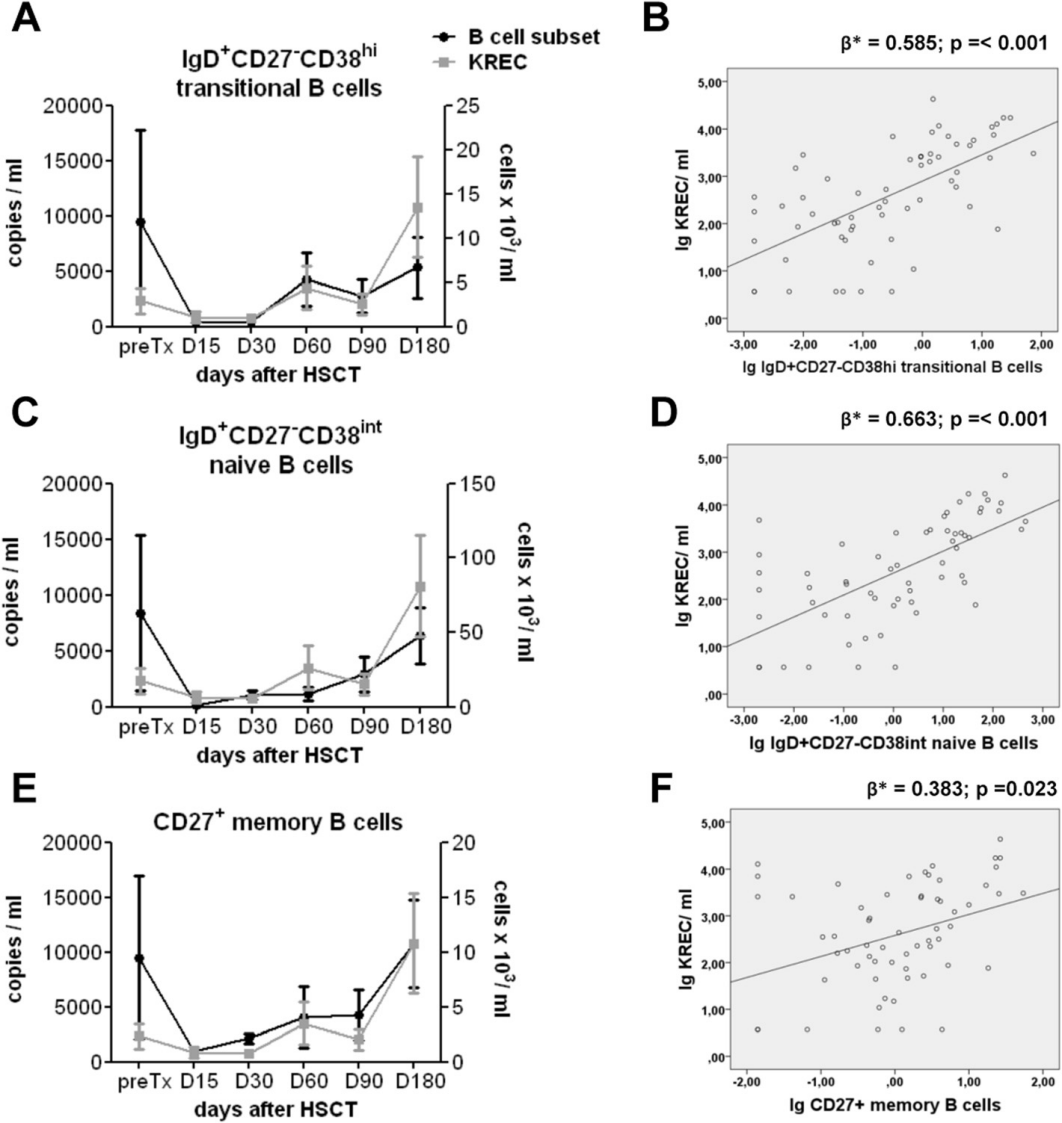
**Correlation analysis of KREC quantification with B-cell subset recovery after alloHSCT. (A,C,E)** Absolute CD19^+^ B-cell subset counts of IgD^+^CD27^-^CD38^hi^ transitional B, IgD^+^CD27^-^CD38^int^ naïve B and CD27^+^ memory B cells and KREC copy number were determined before and after transplantation (preTx: n=7, D15 n=11, D30 n=13, D60 n=8, D90 n=12, D180 n =9). KREC copy number per ml blood (grey line, **A,C,E**) and absolute number of transitional **(A)**, naïve **(C)** and memory B cells **(E)** per ml blood (black line) are shown as mean values ± SEM. **(B,D,F)** Absolute KREC copy number and B-cell subset count are depicted as scatterplots with linear regression line for transitional **(B)**, naïve **(D)** and memory B cells **(F)**. The regression coefficients beta (β*) and p-values are displayed for each subpopulation.

Equivalently, we determined absolute cell counts of IgD^+^CD27^-^CD38^hi^ transitional, IgD^+^CD27^-^CD38^int^ naïve and CD27^+^ memory B cells as well as absolute KREC copy numbers per ml blood before and within six months after transplantation. After transplantation, the number of B cells increased, beginning with memory B cells on day 30 (Figure 3E), transitional B cells on day 60 (Figure 3A), and naïve B cells on day 90 (Figure 3C), but numbers of transitional and naïve B cells remained below pretransplant levels until day 180 (mean 12±10 cells/ml and 63±52 cells/ml, respectively).

Interestingly, already at the pretransplant period we observed a very low number of KRECs with 2331±1155 copies per ml of blood, while a KREC value of 14846 copies per ml was reported for healthy controls (Figure 3A,C,E) [14]. In contrast to TRECs that dropped from higher pretransplant to low posttransplant levels, KRECs only slightly decreased upon transplantation. KRECs started to increase on day 60, and were fivefold higher on day 180 compared to pretransplant level, reaching values of 10809±4510 copies/ml. KREC recovery showed a good and statistically significant correlation with both transitional and naïve B-cell recovery, whereas memory B cells correlated less (Figure 3B,D,F) (ß = 0.585, p < 0.001 transitional; ß = 0.663, p < 0.001 naïve; ß = 0.383, p < 0.05 memory). B cells typically undergo several rounds of cell division during their differentiation into memory cells [23]. Therefore, a low contribution to the KREC pool and consequently less correlation of memory B cells with KRECs was expected. When separating the patients into a full-conditioning and reduced-conditioning group again we still found that KREC levels correlated well with transitional B cells (Additional file 2: Figure S1B). Like for TREC, larger patients cohorts would allow to determine significant differences in ß values for different B cell subsets and different clinical settings.

We further compared the obtained TREC/ KREC values with patients total white blood cell count as measured at each time point in the hospital setting (Additional file 3: Figure S2). TREC as well as KREC kinetics clearly differed from the leukocyte kinetics showing no congruent course of TREC/KREC recovery with leukocyte recovery after alloHSCT. This suggests the TREC/KREC assay to provide useful additional information for monitoring the dynamics of immune system reconstitution after alloHSCT and therefore may have clinical utility. In particular, the duplex PCR presented here is a time- and laboratory costs-saving assay that allows simultaneous quantification of TREC and KREC in only one PCR reaction. TREC/KREC levels are normalized to the quantity of genomic DNA in the sample and variability related to direct DNA quantification is eliminated by using a unique standard curve and TREC/KREC quantification per ml of blood overcomes the issue of peripheral dilution. However, we are aware of the fact, that the measurements alone cannot be considered as direct markers for clinical episodes, because TREC/ KREC can persist in old emigrant cells and disappear after cell death. This easy performable assay rather represents a first-step screening that needs be tested in a larger patient cohort in correlation to clinical parameters, and also should be combined with other methods to help defining the reasons for low TREC/ KREC levels.

## Conclusion

In summary, our study suggests that the combined quantification of absolute TREC and KREC counts is a suitable marker to monitor early T- and B-cell neogenesis in adult patients treated by alloHSCT for hematological malignancies. Both markers exhibited kinetics best comparable with the most primitive T/B cell phenotypes, CD4^+^CD45RA^+^CD31^+^ naïve T-cell as well as transitional (IgD^+^CD27^-^CD38^hi^) and naïve (IgD^+^CD27^-^CD38^int^) B-cell reconstitution, respectively, allowing an estimate of thymic and bone marrow output post alloHSCT. Having validated good correlations, relationship of TREC/ KREC levels and thereby thymic and bone marrow function should be tested next in correlation to clinical episodes such as GvHD, graft failure or Cytomegalovirus and Epstein-Barr virus reactivation in a considerably larger patient cohort allowing multivariate analyses and corrections for confounding effects due to clinical heterogeneity. In case of good correlations with these clinical parameters, TREC and KREC enumeration could be used in combination with T- and B-cell subset quantification in routine clinical monitoring to optimize personalized treatment strategies. For TREC and KREC quantification, low associated reagent costs, DNA stability as well as the need of only small blood volumes would be advantageous especially during lymphocytopenia during the first months after transplantation.

## Competing interests

The authors declare that there are no competing interests.

## Authors’ contributions

AM analyzed results and wrote the manuscript. CO designed and performed RT-PCR experiments and analyzed results. AS performed statistical analysis. FW designed and performed flow cytometry experiments. MS helped analyzing the results. LI provided important material. SF provided important conceptual insights and helped interpreting the results. LU, RA and BD helped interpreting the results. AT and CS provided important conceptual insights and helped interpreting the results. IKN provided important conceptual insights, contributed in experiment design and analysis of results and helped in writing the paper. All authors read and approved the final manuscript.

## Supplementary Material

Additional file 1: Table S1 Specificity controls for the TREC/ KREC quantification assay. Click here for file

Additional file 2: Figure S1 TREC/ KREC level correlation with naïve CD4^+^ T cells/ transitional B cells in patients with full or reduced intensity conditioning therapy. **(A)** Absolute CD4+CD45RA+CD31+ naïve T cell and TREC copy numbers before and after transplantation are shown for patients who received full-conditioning (left) or reduced-conditioning (right) therapy (full-conditioning/reduced conditioning: preTx n=5/2, D15 n=6/5, D30 n=8/5, D60 n=5/3, D90 n=6/7, D180 n=3/5). **(B)** Graphs show absolute transitional B-cell and KREC copy numbers in patients who underwent full-conditioning (left) or reduced-conditioning (right) therapy (full-conditioning/reduced conditioning: preTx: n=5/2, D15 n=6/5, D30 n=8/5, D60 n=6/2, D90 n=6/6, D180 n =3/6). TREC/ KREC copy numbers (grey line) and T-/ B-cell subset number (black line) are displayed as mean values ± SEM. Click here for file

Additional file 3: Figure S2 Incongruent course of absolute TREC or KREC copy counts and leukocyte recovery after alloHSCT. **(A**,**B)** Absolute leukocyte counts were obtained from hospital measurements before and after transplantation (preTx: n=6, D15 n=9, D30 n=11, D60 n=8, D90 n=11, D180 n =4). Shown are mean values ± SEM of TREC **(A)** or KREC **(B)** copy number per ml blood (grey line) and leukocyte count per ml blood (black line, **A**,**B**). Click here for file
