# Supplementary material for: Utilization of TREC and KREC quantification for the monitoring of early T- and B-cell neogenesis in adult patients after allogeneic hematopoietic stem cell transplantation
Source: J Transl Med. 2013 Aug 14;11:188. doi: 10.1186/1479-5876-11-188 (PMC3751290; doi:10.1186/1479-5876-11-188)
Supplement: Additional file 1: Table S1 — Specificity controls for the TREC/ KREC quantification assay. [file 1479-5876-11-188-S1.doc]

**Supplemental Table 1** Specificity controls for the TREC/ KREC quantification assay

|  | PBMC healthy donor Copies in 99 ng DNA | K562 cell line Copies in 100 ng DNA | Jurkat cell line Copies in 61 ng DNA |
| --- | --- | --- | --- |
| TREC copies | 22 | undetermined | undetermined |
| KREC copies | 126 | undetermined | undetermined |
| TRAC copies | 25545 | 24901 | 13773 |
