# Supplementary material for: Utilization of TREC and KREC quantification for the monitoring of early T- and B-cell neogenesis in adult patients after allogeneic hematopoietic stem cell transplantation
Source: J Transl Med. 2013 Aug 14;11:188. doi: 10.1186/1479-5876-11-188 (PMC3751290; doi:10.1186/1479-5876-11-188)
Supplement: Additional file 2: Figure S1 — TREC/ KREC level correlation with naïve CD4+ T cells/ transitional B cells in patients with full or reduced intensity conditioning therapy. (A) Absolute CD4+CD45RA+CD31+ naïve T cell and TREC copy numbers before and after transplantation are shown for patients who received full-conditioning (left) or reduced-conditioning (right) therapy (full-conditioning/reduced conditioning: preTx n=5/2, D15 n=6/5, D30 n=8/5, D60 n=5/3, D90 n=6/7, D180 n=3/5). (B) Graphs show absolute transitional B-cell and KREC copy numbers in patients who underwent full-conditioning (left) or reduced-conditioning (right) therapy (full-conditioning/reduced conditioning: preTx: n=5/2, D15 n=6/5, D30 n=8/5, D60 n=6/2, D90 n=6/6, D180 n =3/6). TREC/ KREC copy numbers (grey line) and T-/ B-cell subset number (black line) are displayed as mean values ± SEM. [file 1479-5876-11-188-S2.doc]

***Supplemental Figure 1 TREC/ KREC level correlation with naïve CD4^+^ T cells/ transitional B cells in patients with full or reduced intensity conditioning therapy. (A)*** *Absolute CD4+CD45RA+CD31+ naïve T cell and TREC copy numbers before and after transplantation are shown for patients who received full-conditioning (left) or reduced-conditioning (right) therapy (full-conditioning/reduced conditioning: preTx n=5/2, D15 n=6/5, D30 n=8/5, D60 n=5/3, D90 n=6/7, D180 n=3/5).* ***(B)*** *Graphs show absolute transitional B-cell and KREC copy numbers in patients who underwent full-conditioning (left) or reduced-conditioning (right) therapy (full-conditioning/reduced conditioning: preTx: n=5/2, D15 n=6/5, D30 n=8/5, D60 n=6/2, D90 n=6/6, D180 n =3/6). TREC/ KREC copy numbers (grey line) and T-/ B-cell subset number (black line) are displayed as mean values ± SEM.*
