# Supplementary material for: Utilization of TREC and KREC quantification for the monitoring of early T- and B-cell neogenesis in adult patients after allogeneic hematopoietic stem cell transplantation
Source: J Transl Med. 2013 Aug 14;11:188. doi: 10.1186/1479-5876-11-188 (PMC3751290; doi:10.1186/1479-5876-11-188)
Supplement: Additional file 3: Figure S2 — Incongruent course of absolute TREC or KREC copy counts and leukocyte recovery after alloHSCT. (A,B) Absolute leukocyte counts were obtained from hospital measurements before and after transplantation (preTx: n=6, D15 n=9, D30 n=11, D60 n=8, D90 n=11, D180 n =4). Shown are mean values ± SEM of TREC (A) or KREC (B) copy number per ml blood (grey line) and leukocyte count per ml blood (black line, A,B). [file 1479-5876-11-188-S3.doc]

***Supplemental Figure 2* Incongruent course of absolute TREC or KREC copy counts and leukocyte recovery after alloHSCT. (A,B**) Absolute leukocyte counts were obtained from hospital measurements before and after transplantation (preTx: n=6, D15 n=9, D30 n=11, D60 n=8, D90 n=11, D180 n =4). Shown are mean values ± SEM of TREC (**A**) or KREC (**B**) copy number per ml blood (grey line) and leukocyte count per ml blood (black line, **A,B**).
